# *Plasmodium falciparum* cGMP-Dependent Protein Kinase – A Novel Chemotherapeutic Target

**DOI:** 10.3389/fmicb.2020.610408

**Published:** 2021-02-03

**Authors:** David Rotella, John Siekierka, Purnima Bhanot

**Affiliations:** ^1^Department of Chemistry and Biochemistry, Montclair State University, Montclair, NJ, United States; ^2^Department of Microbiology, Biochemistry and Molecular Genetics, Rutgers New Jersey Medical School, Newark, NJ, United States

**Keywords:** *Plasmodium*, malaria, cGMP signaling, second messenger, kinase

## Abstract

The primary effector of cGMP signaling in *Plasmodium* is the cGMP-dependent protein kinase (PKG). Work in human-infective *Plasmodium falciparum* and rodent-infective *Plasmodium berghei* has provided biological validation of *P. falciparum* PKG (PfPKG) as a drug target for treating and/or protecting against malaria. PfPKG is essential in the asexual erythrocytic and sexual cycles as well as the pre-erythrocytic cycle. Medicinal chemistry efforts, both target-based and phenotype-based, have targeted PfPKG in the past few years. This review provides a brief overview of their results and challenges.

## PKG Is the Key Mediator of cGMP Signaling in *Plasmodium*

cGMP is a second-messenger in eukaryotic cells. It is synthesized from GTP by guanylate cyclases and hydrolyzed by phosphodiesterases. Changes in intracellular levels of cGMP are converted to cellular responses through the action of effector proteins such as cGMP-dependent protein kinases [also known as protein kinase G (PKG)], cGMP-gated ion channels and cGMP binding proteins. *Plasmodium* genomes encode single copies of PKG and a putative cGMP-binding protein but the latter lacks key residues required for cyclic nucleotide binding (Moon et al., 2009). There are no homologs of cGMP-gated ion channels in the genome. PKG is likely to be the key mediator of cGMP signaling in the parasite.

cGMP-dependent protein kinase consists of a carboxy terminus regulatory domain fused to a kinase domain. The regulatory domain of *Plasmodium* (and other Apicomplexan) PKGs contain four cGMP binding sites, with one of them being degenerate and incapable of binding cGMP. In contrast mammalian PKGs have two cGMP binding sites. Another difference between mammalian and *Plasmodium* PKGs is while the former dimerizes, the latter is found as a monomer. Mammalian PKG is regulated through the combined action of an autoinhibitory segment present in the kinase’s amino domain and by cGMP binding. In conditions of low cGMP, its substrate site is occupied by an autoinhibitory segment (Wall et al., 2003; Alverdi et al., 2008). Increasing cGMP levels lead to allosteric and cooperative occupation of the cGMP-binding sites in the regulatory domain, lifting the autoinhibition and activating the kinase domain. The kinase then phosphorylates substrate proteins on Ser or Thr residues. Regulation of *Plasmodium* PKG also requires cGMP binding (Kim et al., 2015; El Bakkouri et al., 2019; Byun et al., 2020) but, in a difference from mammalian PKG, not its putative autoinhibitory segment (Franz et al., 2018). PKG-dependent phosphorylation was detected in almost a 100 proteins in *P. falciparum*’s erythrocytic stages (Alam et al., 2015; Vanaerschot et al., 2020) and in 193 proteins in *P. berghei* ookinetes (Brochet et al., 2014). The diversity of substrates indicates the variety of cellular pathways regulated by PKG. Interestingly, *P. falciparum* PKG (PfPKG) has a substrate-site preference that is substantially different from its mammalian homolog (Govindasamy et al., 2019).

## PKG Functions in Egress of Erythrocytic Merozoites

Conditional and chemical genetics have established the essential role of PKG in the asexual cycle (Taylor et al., 2010), specifically in the exit of merozoites from schizonts (Kim et al., 2015; Ganter et al., 2017). In *P. falciparum* schizonts PKG regulates the timely release of the protease SUB1 from exonemes into the parasitophorous vacuole and of AMA1 from micronemes to the merozoite surface (Collins et al., 2013). The net result of inhibiting PfPKG is a block in merozoite egress and interruption of the asexual cycle. The underlying mechanism of PfPKG’s action is its regulation of phosphoinositide metabolism and consequently Ca^2+^ mobilization in the parasite (Brochet et al., 2014; recently reviewed in Brochet and Billker, 2016). In the related Apicomplexan, *Toxoplasma gondii* there is evidence that PKG-regulated egress of parasites is antagonized by cAMP signaling mediated by the parasite’s cAMP dependent protein kinase pathways (Jia et al., 2017). Chemical inhibition of *T. gondii* PKG blocks parasite egress induced through genetic downregulation of *T. gondii* PKA signaling. Similar interplay between PKG and PKA pathways in *Plasmodium* has not yet been reported although *P. falciparum* PKA is essential for merozoite invasion (Wilde et al., 2019).

## PKG Is Required for Gametocyte Activation and Ookinete Motility

In the mosquito midgut, activation of *Plasmodium* gametocytes to form gametes requires PKG. Its inhibition prevents the rounding up of gametocytes, an early step in gametocyte activation (McRobert et al., 2008). In mature ookinetes, PKG function is required for motility a prerequisite to ookinete invasion of the midgut (Brochet et al., 2014). As in asexual stages, in gametocytes and ookinetes PKG mobilizes intracellular Ca^2+^ and regulates vesicular traffic (Brochet et al., 2014). PKG-dependent phosphorylation of proteins that are part of the actinomyosin motor also likely contributes to its regulation of parasite motility (Brochet et al., 2014; Govindasamy et al., 2019).

## PKG Is Essential for Parasite Invasion of and Exit From Hepatocytes

Conditional and chemical genetic approaches demonstrated that PKG plays a dual role in the pre-erythrocytic cycle. It is required for sporozoite motility and hence their invasion of hepatocytes for as well as for the formation and/or release of merosomes from infected hepatocytes. PKG’s effect on sporozoite motility is mediated through the release onto the sporozoite surface of micronemal adhesins, such as TRAP (Govindasamy et al., 2016), which enable parasite attachment to the extracellular surface. Later during liver stage development, chemical inhibition of PKG or depletion of the protein prevents the formation and/or release of merosomes, membrane-bound packets of hepatic merozoites that are extruded from the infected hepatocyte to initiate the transition from the liver cycle to the erythrocytic cycle (Falae et al., 2010; Govindasamy et al., 2016; Govindasamy and Bhanot, 2020).

## Targeting *P. falciparum* PKG in the Asexual Cycle

The essential function of PfPKG in schizogony provided biological validation for its development as a drug target for treating the pathology-causing erythrocytic stages of *P. falciparum*. It is a new target in the global malaria drug discovery landscape. Inhibitors of PfPKG represent a novel mechanism of action since, based on literature precedent, none of the current compounds under development target PfPKG (Wells et al., 2015). Therefore, cross-resistance with existing agents is less likely. Medicinal chemistry programs directed against PfPKG have focused on activity against asexual stages in the blood and gametocytes in mosquitoes (Matralis et al., 2019; Penzo et al., 2019).

*Plasmodium* PKG is significantly different from mammalian PKG to allow specific targeting. A trisubstituted pyrrole, (**1**) (also called TSP) is a potent and selective inhibitor of PKGs of several Apicomplexan parasites (Diaz et al., 2006). It has an IC_50_ of ∼3.5 nM against PfPKG (Diaz et al., 2006; McRobert et al., 2008) and 2 μM against human PKG (our unpublished data). This significant selectivity is associated with a larger “gatekeeper” residue, Gln in human PKG (Gurnett et al., 2002). PfPKG contains Thr at the “gatekeeper” position (amino acid 618) and its shorter side-chain allows access to compounds excluded from the smaller cavity in the human enzyme. Replacement of Thr618 in PfPKG with Gln (T618Q) leads to a 3000-fold increase in IC_50_ of the mutant enzyme against **1** (McRobert et al., 2008). Merck researchers pursued **1** and the imidazopyridine **2** as lead compounds for a veterinary medicine campaign directed against PKG of a related parasite, *Eimeria tenella* (Biftu et al., 2005, 2006). They succeeded in identifying lead subnanomolar compounds in both the TSP series, **1** and the imidazopyridine series, **2** but the program was ultimately discontinued due to Ames positive data and genotoxicity of the two top imidazopyridine compounds (Biftu et al., 2006). An imidazo (Wall et al., 2003; Moon et al., 2009) thiazole series was subsequently investigated as an alternative. This research took advantage of established structure activity information from previous work by this group that identified optimal 4-fluorophenyl and aminopyrimidine substituents on the heterocycle. A narrow range of amide- and amino-derived possibilities was examined at the remaining thiazole position. In this group, amines were substantially more potent compared to the corresponding amide analog. This identified a derivative (**3**) with potent *in vitro* PKG activity (IC_50_ 80 pM) and broad-spectrum *in vivo* potency in anticoccidial assays (Scribner et al., 2008).


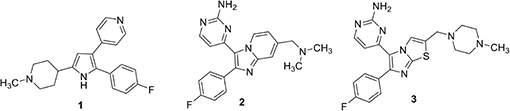


Drug discovery and PfPKG inhibitor optimization will be assisted by the availability of crystal structures of both *P. falciparum* and *Plasmodium vivax* PKG (El Bakkouri et al., 2019). This structure of the apoprotein showed how regions of the protein are arranged in the inactive structure. Based on this information, the team proposed a mechanism for activation by cGMP binding that may prove useful in further biochemical studies.

In an important paper that provided direct preclinical validation of PfPKG as a target for antimalarial therapy, Baker et al. (2017) reported that an imidazopyridine based PfPKG inhibitor could clear *P. falciparum* infection in mice engrafted with human erythrocytes. This molecule, **4** (ML10), resulted from a structure-activity study that was aided by structure-based design. Specific hydrogen bonding interactions between the sulfonamide substituent in the pendant aryl ring with Asp675 and Phe670 in the enzyme, along with key hinge region interactions between Val614 and the pyrimidine moiety contributed to excellent *in vitro* potency (IC_50_ 160 pM) that translated very well to cell based studies (EC_50_ 2 nM) and high selectivity in a panel of human kinases. As expected, ML10 was several orders of magnitude less potent *in vitro* against the “gatekeeper” mutant PfPKG (T618Q) and similarly less active in culture of asexual stage parasites expressing the mutant enzyme. Oral administration of **4** to mice at a dose of 25 mg/kg twice daily reduced *P. berghei* parasitemia by 50–60% over 4 days. A single 50 mg/kg dose of **4** reduced *Plasmodium chabaudi* infection by approximately 50% in mice and two 50 mg/kg doses in one day was as effective as a single 10 mg/kg dose of chloroquine in this model. Greater efficacy against *P. chabaudi* was attributed to its synchronized asexual cycle, because of which the twice-daily dosing was able to span the entire merozoite egress-reinvasion window. Using blood stage *P. falciparum* in mice, **4** was dosed at 50 or 100 mg/kg for 4 days, reduced parasitemia substantially in a dose response manner. Interestingly, attempts to generate *P. falciparum* strains resistant to **4** in culture were unsuccessful. Other cell culture experiments showed that **4** had no cytotoxic effect on three different human cell lines at concentrations as high as 10 μM.


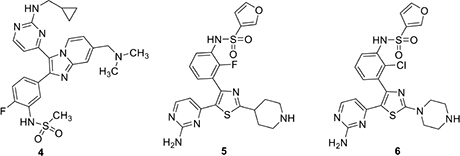


Penzo et al. (2019) carried out a high throughput screen of a 1.7 million compound library provided by GlaxoSmithKline. This screen identified a number of hit scaffolds that included imidazoles, pyrazoles, and thiazoles. The group chose to focus on thiazoles because previous experience suggested this template offered the greatest potential for improved selectivity among human kinases. Chemoproteomic experiments with thiazole examples identified in the screen revealed that in addition to potent PfPKG inhibition (IC_50_ ∼ 1 nM), some of these compounds also inhibited CDPK1, NEK1, CDPK4, CK1, and CRK5. In a 48-h *P. falciparum* growth inhibition assay, two of these compounds, **5** and **6**, gave good activity, indicative of a desired fast killing profile. It was equally interesting to observe that these compounds were equally active against *P. falciparum* parasites that expressed a T618Q mutant PfPKG. The hits were substantially less potent *in vitro* against the mutant enzyme, suggesting that these compounds are acting at one or more other targets in the parasite.

The Baker group reported progress in the optimization of thiazoles in 2018 and interest in them may have been stimulated by a combination of the Ames results and potential for good kinase selectivity noted above (Tsagris et al., 2018). The team applied established structure-activity information associated with **1** as a starting point for their work. The monosubstituted pyridine in **1** is a likely site for metabolism limiting oral bioavailability and as a result, alternatives were sought. An aminopyrimidine replacement **7** was identified with good *in vitro* PfPKG potency (IC_50_ 17 nM), but measurably weaker cellular activity (EC_50_ ∼1800 nM), compared to **1**. Substituents were added to the primary amino group and this resulted in phenyl piperazine derivative **8** with improved PfPKG activity (IC_50_ 300 pM) and excellent cellular activity (EC_50_ 25 nM). Using the binding model developed earlier, it was hypothesized the phenyl piperazine filled a hydrophobic pocket more completely. However, this compound was not as selective as **6** and modifications to the 4-fluorophenyl moiety were evaluated. This resulted in identification of **9**, a highly selective PfPKG inhibitor with slightly lower cellular efficacy compared to **8** (EC_50_ 115 nM).


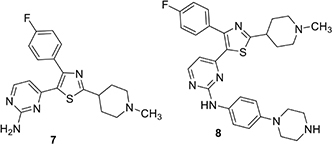


More recently, the Baker group disclosed the identification of novel trisubstituted thiazoles that exhibited rapid parasite killing properties (Matralis et al., 2019). There is a need in the field to develop such rapid killing compounds because of increasing resistance to artemisin-class antimalarials. This article revealed that a highly selective PfPKG inhibitor such as **9** exhibited slow parasite killing kinetics. The team focused on pyrimidinyl nitrogen substituents because previous experience showed that this position played a key role in PfPKG potency. This therapeutic hypothesis stimulated the team to focus primarily on whole-cell activity rather than potency against PfPKG and, following development principles, kept other important pharmaceutical properties in mind such as hERG, cytotoxicity, metabolic clearance rate and water solubility. Cell culture experiments measured kinetic effects on *P. falciparum* killing and new compounds were compared using artesunate (an artemisinin analog), pyrimethamine and atovaquone as positive controls to provide kinetic range in the assay. Transmission blocking activity was measured by assessment of exflagellation of male gametocytes and by expression of Pfs25 protein expression in female gametocytes. There is substantial evidence that PfPKG plays an essential role in gametogenesis. Two compounds were highlighted in this paper, **10** and **11**. While **10** had unfavorable hERG activity (IC_50_ 1 μM), it rapidly killed *P. falciparum* in culture, exhibiting a rate and extent (>90% at 24 h) comparable to artesunate. In addition, **10** blocked transmission with EC_50_ values of 300 and 400 nM, respectively in male and female gametocyte assays. Furthermore, **10** was equally efficacious when tested in culture using wild-type and T618Q mutant *P. falciparum* with EC_50_ values approximately 150 nM. Further SAR led to the identification of **11** with comparable fast killing properties and lower hERG activity (IC_50_ 4 μM). Compound **10** was employed in chemoproteomic experiments to identify other proteins that might either contribute to its rapid killing activity and/or provide new targets for future investigation. These experiments highlighted a serine/arginine protein kinase, SRPK2, as a candidate for further investigation.


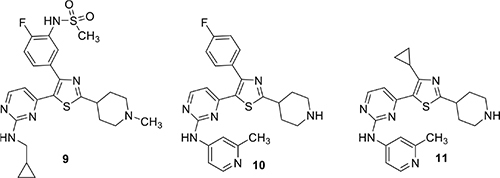


Another compound, MMV030084 **(12)** targeting PfPKG was found through phenotypic screening using sporozoite invasion and asexual stage growth assays (Vanaerschot et al., 2020). It also blocks male gametogenesis. Chemoproteomics was then used to determine that **12** interacts with PfPKG. Conditional depletion of PfPKG protein in *P. falciparum* asexual parasites decreased the IC_50_ of the compound by fourfold, consistent with heightened sensitivity of the parasite to a compound when the compound’s *in vivo* target is less abundant. Compound **12** is structurally similar to **1** and molecular docking studies suggest that it exploits the same binding pocket.


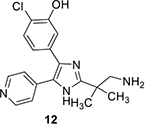


## Targeting PfPKG for Chemoprotection

The “Chemoprotection” against malaria target product profile is described as a combination of two or more drugs that are active against *Plasmodium*’s hepatic schizonts and may also have activity against asexual blood stages (Burrows et al., 2017). Our data provide proof-of-principle that inhibition of the pre-erythrocytic cycle through specific and selective chemical targeting of *Plasmodium* PKG is possible, making it a target for development of chemoprotective drugs.

We have demonstrated that **1** targets *P. berghei* PKG (PbPKG) and blocks protein secretion and therefore parasite motility, invasion of hepatocytes and formation of liver stages by *P. berghei* sporozoites (Govindasamy et al., 2016). Treatment with **1** post-invasion leads to liver stages that are significantly smaller in size (our unpublished data). It also blocks maturation of liver schizonts into merosomes, membrane-bound packets of hepatic merozoites that are extruded from an infected hepatocyte into the bloodstream (Govindasamy et al., 2016). Liver stages that are developmentally arrested due to loss of PbPKG elicit a protective host immune response that protects from future infection by wild-type sporozoites (Falae et al., 2010). The block in merosome formation and/or release resulting from **1**’s inhibition of PbPKG (Govindasamy et al., 2016) is similar to the phenotype of *P. berghei* liver stages mutants missing PKG (Falae et al., 2010).

The EC_50_ of **1** against transgenic *P. berghei* sporozoites and liver stages expressing the “gatekeeper” PbPKG (T_619_Q) is significantly higher than against wild-type sporozoites (EC_50_ = 2.12 vs. 0.11 μM) (Govindasamy et al., 2016). The decreased sensitivity of T_619_Q-PKG sporozoites and liver stages to **1** demonstrates that its target in pre-erythrocytic stages is PKG, and that PKG is essential for sporozoite infection and liver stage maturation. Importantly, mice treated with **1**, before and shortly after sporozoite infection, experience a significant delay or abolishment in blood stage parasitemia (Panchal and Bhanot, 2010).

This work also clarified the seeming contradiction between PbPKG being **1**’s primary target in sporozoites and our observations that *P. berghei* mutant sporozoites in which the PbPKG gene has been conditionally deleted infect HepG2 cells normally and remain sensitive to **1** (Falae et al., 2010). We demonstrated that conditional deletion of PbPKG in sporozoites does not significantly decrease the long-lived PbPKG protein (Govindasamy et al., 2016). The near-normal levels of PbPKG protein in mutant sporozoites enable normal infectivity and sensitivity to **1**.

The pre-erythrocytic cycle of *Plasmodium* could be particularly vulnerable to *Plasmodium* PKG inhibitors because the compounds would have two opportunities to block the cycle. The first block will prevent sporozoite infection of the liver. Parasites that escape the first block will be targeted during their 1–2 week long intrahepatic period by a second block – inhibition of the development/exit of mature liver forms from the hepatocyte. Developmentally arrested liver stages will be targeted by the host immune response that can clear them, and potentially provide protection against subsequent infections. We believe that these aspects of PKG function, the significantly lower parasite load in the liver, and the single infection cycle in the liver make pre-erythrocytic stages vulnerable to *Plasmodium* PKG inhibitors. In addition, *Plasmodium* PKG inhibitors will target the erythrocytic cycle and transmission to mosquitoes since the kinase is essential for merozoite egress and invasion, and for gametogenesis (Baker et al., 2017).

## Assays for PfPKG Inhibition

*In vitro* activity of PfPKG has been assayed using a variety of formats including microfluidic mobility shift assays (Baker et al., 2017), immobilized metal ion affinity-based fluorescence polarization (IMAP) assays (Mahmood et al., 2020) and an NADH/ATPase coupled assay (El Bakkouri et al., 2019). In the microfluidic mobility shift assay, kinase reactions are performed using a fluorescently labeled peptide substrate. Phosphorylated products are separated, based on charge differences, by capillary electrophoresis and detected by laser-induced fluorescence. This technology allows acquisition of enzymatic data in a kinetic fashion which enables simultaneous measurement of both product and substrate concentrations for each reaction. The IMAP technology is based on the specific, high-affinity interaction of trivalent metal-containing nanoparticles with phosphorylated substrates. During the course of a kinase reaction fluorescently labeled substrate is phosphorylated and binds to metal nanoparticles, resulting in a change in fluorescence polarization between free peptide and nanoparticle-associated phosphorylated peptide, a direct measure of kinase activity. The NADH/ATPase coupled assay is a photometric microtiter plate assay which measures the accumulation of ADP during a kinase reaction and is based on ADP recycling coupled to the oxidation of NADH. One advantage of this assay format is that it does not require fluorophore labeled substrates. All three assays lend themselves to high throughput screening and can be used for both kinetic analysis (for example to determine a compound’s mode of inhibition) and endpoint analysis (single determination at a defined time point). The IMAP and NADH/ATPase coupled assays are single step or “homogeneous” assays with no requirement for separation of reaction products. All three assays were so far deployed at the K_m_ of ATP and hence selected for ATP-competitive inhibitors of PfPKG. It is interesting to speculate that compound screens executed at saturating concentrations of ATP might identify compounds with novel modes of inhibition such as allosteric inhibitors of PfPKG.

Exploiting screening strategies to identify non-competitive or allosteric inhibitors could lead to the identification of novel inhibitors with enhanced selectivity. Screening compound libraries at high substrate concentrations for example, could bias a screen toward identifying non-competitive inhibitors. Other screening technologies such as ThermoFluor (Zhang and Monsma, 2010) could be utilized to identify non-competitive inhibitors. ThermoFluor or differential scanning fluorimetry (DSF) assesses the direct binding of a ligand to its target protein by measuring the enhancement of the thermal stability of the target protein. The technique provides real-time analysis of ligand (compound) binding to a target protein. The structural differences between PfPKG and the human ortholog (number of cGMP binding domains and the role of the pseudosubstrate autoinhibitory sequence) may provide a conformational arrangement favorable to identifying unique allosteric inhibitory sites. ATP non-competitive inhibitors have been successfully identified for a variety of protein kinases such as the Bcr Abl kinase inhibitor, Gleevec which was the first successful therapy for chronic myelogenous leukemia (Druker et al., 1996). Recent work has demonstrated the potential for allosteric inhibition of PfPKG by targeting its key cGMP binding site (Byun et al., 2020).

The activity of PfPKG inhibitors in whole cells has been assayed in asexual stages, gametocytes and liver stages. For determining the effect on *P. falciparum* asexual stage growth, inhibitors are added to cultures composed primarily of ring-stages (drug-sensitive 3D7 strain) at *t* = 0 h (Desjardins et al., 1979; Baker et al., 2017). Radioactive hypoxanthine is added to cultures at 48 h, which corresponds to the time of parasite release from schizonts, and its incorporation into parasites is measured at 72 h. This assay captures the effect of compounds on parasite growth in a 24 h cycle.

The *in vivo* efficacy of PfPKG inhibitors against asexual stages has been tested using *P. berghei* ANKA, *P. chabaudi* and a *P. falciparum*-humanized mouse model (Baker et al., 2017). Mice infected with *P. berghei* ANKA or *P. chabaudi* were treated with compounds for 4 consecutive days. Since *P. chabaudi* undergoes a synchronized intra-erythrocytic cycle, compound administration was timed to coincide with schizont rupture and merozoite reinvasion of RBCs. Parasitemias of treated and control mice were determined after 4 days of treatment. *In vivo* efficacy against *P. falciparum* asexual stages was determined in the GSKPfalcHuMouse (Jimenez-Diaz et al., 2009). These immunodeficient mice are engrafted with human erythrocytes to achieve 40% chimerism and then infected with *P. falciparum* 3D7 asexual stage parasites. Compound administration commences 3 days post-infection and parasitemias are determined after 4 days of consecutive treatment.

Effect of PfPKG inhibitors on male gametocytes has been determined by measuring exflagellation centers observed upon addition of xanthurenic acid to a NF54 gametocyte-containing culture incubated at about 27°C (Delves et al., 2013; Matralis et al., 2019). Activation of female gametocytes was assayed in the same culture using an antibody against Pfs25, a protein expressed on the surface of female gametes (Delves et al., 2013; Matralis et al., 2019). The effect of these inhibitors on parasite development in mosquitoes is assayed by microscopic counting of oocysts present in midguts of mosquitoes at day 7 post-feeding of a gametocyte bloodmeal (Baker et al., 2017).

Determining the efficacy of PfPKG inhibitors against pre-erythrocytic stages has utilized rodent-infective *P. berghei* ANKA sporozoites. Sporozoites obtained from salivary glands of infected mosquitoes are added to HepG2 cells, a cultured human hepatoma cell line, together with inhibitors. Inhibitor-containing medium is replaced with inhibitor-free medium at 14–18 h post-infection. The number of infected HepG2 cells is determined at 40–48 h post-infection using immunostaining with a parasite-specific antibody or measurement of luciferase activity if luciferase-expressing sporozoites were used. Therefore this assay measures the effect of PfPKG inhibition on sporozoite entry into hepatocytes (Govindasamy et al., 2016). The *in vivo* efficacy of this inhibition was examined using *P. berghei* and *Plasmodium yoelii*-infected mice (Panchal and Bhanot, 2010). Sporozoites were delivered through either mosquito bite or intravenous injection. Compounds were administered prior to sporozoites and 2 h post-infection to cover a time-window spanning sporozoite exit from the site of bite and their invasion of the liver. Parasite burden in the liver was determined, at 40 h post-infection, by quantifying either parasite 18S rRNA expression via real-time PCR or bioluminescence in the liver, if using luciferase-expressing sporozoites, via *in vivo* intravital imaging (IVIS). The length of time between sporozoite inoculation and appearance of parasites in the blood (pre-patent period) can also used as an indirect measure of liver-cycle inhibition.

## Safety Considerations for Targeting *Plasmodium* PKG

Targeting kinases requires an extra consideration for toxicity. This risk can be minimized through rigorous incorporation of structural features that optimize affinity for the *P. falciparum* kinase and reduce affinity for human kinases. Development of top compounds requires human kinome-wide selectivity analysis to ensure selectivity and enhance safety. In a chemoprotective role, PfPKG inhibitors will be administered for weeks to a few months, rather than chronically as in some other disease indications that use kinase inhibitors, further minimizing the risk from potential off-target activity of a parasite kinase-targeted molecule.

## Parasite Resistance to PfPKG Inhibitors

Two studies have investigated the potential for parasites to evolve resistance to PfPKG inhibitors. In (Baker et al., 2017), exposure of parasites cultures to sub-lethal doses of **2** (3× IC_50_) for 60 days did not lead to selection of resistance. In (Vanaerschot et al., 2020), parasites were similarly subjected to sub-lethal doses of **12** and parasites with low-level resistance were selected. Whole-genome sequencing of resistant parasites identified a mutation in the Tyrosine-kinase like protein 3 (TKL3) but none in PfPKG. Introduction of this mutation into WT parasites or knocking-out TKL3 caused a threefold shift in the IC_50_ for **12**. However, **12** did not inhibit the activity of recombinant TLK3 *in vitro*, even at 500× IC_50_. These data strongly suggest that TKL3 mediates resistance against **12** and is not its target *in vivo*. It is proposed that phosphorylation of an essential PfPKG substrate by TKL3 permits the parasite to escape the egress defect imposed by inhibition of PfPKG by **12**.

## Other *P. falciparum* Cyclic Nucleotide-Dependent Protein Kinases as Potential Drug Targets

*Plasmodium falciparum* contains a single cAMP-dependent protein kinase (PfPKA), composed of catalytic and regulatory subunits. PfPKA has been shown to phosphorylate *P. falciparum* apical membrane antigen 1 (PfAMA1) located in the micronemes of *P. falciparum* merozoites and is critical for parasite invasion (Patel et al., 2019; Wilde et al., 2019). Asexual stages of parasites lacking PfPKA exhibit a profound reduction in growth along with a reduction in erythrocyte invasion. The interplay between PfPKG and PfPKA in liver invasion by sporozoites remains to be established and may have important therapeutic implications.

## Author Contributions

PB, JS, and DR wrote and edited the manuscript. DR provided the figures. All authors contributed to the article and approved the submitted version.

## Conflict of Interest

The authors declare that the research was conducted in the absence of any commercial or financial relationships that could be construed as a potential conflict of interest.
